# The COMEBACK Study: Social Determinants of Health Impact on Virologic Suppression in a 48-Week Low-Barrier-Care Study of Rapid Antiretroviral Therapy Reinitiation Among Persons With HIV Lost to Care

**DOI:** 10.1093/ofid/ofag216

**Published:** 2026-04-18

**Authors:** Gregory D Huhn, Rebecca Osborn, Lindsey Roden, Kody Keckler, Camille DeMarco, Patricia Cortes Valadez

**Affiliations:** The Ruth M. Rothstein CORE Center, Chicago, Illinois, USA; Cook County Health, Chicago, Illinois, USA; The Ruth M. Rothstein CORE Center, Chicago, Illinois, USA; Cook County Health, Chicago, Illinois, USA; The Ruth M. Rothstein CORE Center, Chicago, Illinois, USA; Cook County Health, Chicago, Illinois, USA; The Ruth M. Rothstein CORE Center, Chicago, Illinois, USA; The Ruth M. Rothstein CORE Center, Chicago, Illinois, USA; The Ruth M. Rothstein CORE Center, Chicago, Illinois, USA

**Keywords:** Biktarvy, HIV, low-barrier care, social determinants of health, virologic suppression

## Abstract

**Background:**

Effectively preventing transmission is critical to ending the HIV epidemic (EHE). The primary objective of the COMEBACK study, a 48-week single-center study, was to reengage lost-to-care persons with HIV and rapidly reinitiate antiretroviral therapy (ART) with bictegravir/emtricitabine/tenofovir alefenamide (B/F/TAF) in a low-barrier-care model to promote virologic suppression (VS).

**Methods:**

Adults off ART for ≥2 weeks, without significant B/F/TAF resistance or renal impairment, were started on B/F/TAF upon reengagement after same-day baseline labs (n = 100). Participants self-screened into case management (CM) tiers: minimal, moderate, or advanced. Participants requiring additional support (tier escalation) were identified during the study. The associations of baseline characteristics and 5 social determinants of health (SDoH) assessments with VS status at study end were analyzed.

**Results:**

At baseline, the median age was 37 years, with 90% Black and 68% cisgender male. Median CD4+ was 310 cells/mm^3^, with a median viral load of 11 084 copies/mL (16% VS). Median time off ART was 5 months. Fifty-nine of 100 participants required tier escalation. Sixty-six of 100 participants were retained in care at 48 weeks, with VS (HIV-1 RNA <200 copies/mL) in 54% of the intent-to-treat population and 82% (n = 54/66) of the observed population. One SDoH (adherence concerns) was significantly associated with non-VS. No resistance to B/F/TAF was detected through 48 weeks.

**Conclusions:**

VS was high for participants with rapid ART reinitiation retained in care. CM escalation and baseline adherence concerns were associated with non-VS at study end. Achieving VS among high-risk populations disenfranchised from care will likely require further innovation in intense individualized CM and retention approaches to capitalize on low-barrier-care models toward EHE.

## Teaser

Virologic suppression was achieved in 82% of participants retained in care at week 48, approaching national rates for all persons with HIV (PWH) in Ryan White clinics, with a majority of participants requiring escalation of case-management resources due to social determinants of health challenges and lapses in retention occurring among one-third of participants by study end, predominantly driven by disrupted smartphone communications. The findings demonstrate that achieving virologic suppression among high-risk PWH populations disenfranchised from care will likely require not only individualized case management and specialized retention approaches but integrated sustainable strategies among multiple levels of social determinants of health interventions to advance low barrier care models.

In February 2019, a federal proposal was released to end the HIV-1 epidemic (EHE) and new infections in the United States in the next 10 years [1]. Of the 1.2 million estimated people with HIV (PWH) in the United States, only 54% are in continuous care, with 66% having sustained virologic suppression (VS) [2]. Modeling studies support increasing VS among PWH to reduce HIV transmission in the United States [3, 4].

Cook County in Illinois is 1 of the 48 counties with the highest burden of HIV transmissions in the United States [1]. PWH who’ve been diagnosed but are not retained in care are estimated to account for the largest proportion of HIV transmissions; identifying and effectively targeting this population is key to mitigating new infections [5]. Risk factors for poor engagement and retention include poverty, housing insecurity, lapses in insurance or access to primary care, substance use disorders, and mental illness [6–8].

Proactive reengagement of PWH who miss appointments and/or are lost to follow-up is recommended through strengths-based case management (CM), intensive outreach, partnering with AIDS service organizations, and, in some settings, financial incentives for antiretroviral therapy (ART) reinitiation [9–12]. The recent HPTN 078 trial identified PWH out of care in the United States and randomized them to either standard of care or enhanced CM for linkage/treatment for 1 year. There was no statistical difference in VS rates, suggesting that individual-level CM to address specific barriers to care is necessary [13].

The Ruth M. Rothstein (RMR) CORE Center is a large, urban, safety-net HIV clinic that cares for ∼5000 PWH in Cook County. The retention-in-care rate, defined as at least 2 visits or 2 HIV-1 RNA VL reports occurring at least 3 months apart within the year, was 75% in 2018.

Often when out-of-care PWH who are off ART reengage with clinical providers, routine labs are collected (including testing HIV resistance to ART when indicated), health care benefits are reassessed, and the most recent ART regimen is restarted if the PWH agrees to treatment. Characteristics of prior regimens may present barriers to adherence or perform suboptimally in PWH with certain immunologic and/or viral factors, possibly affecting VS, including multiple pills, drug–drug interactions, variable tolerability, low CD4+ T-cell counts, high HIV-1 RNA VL, or low thresholds for resistance.

Bictegravir/emtricitabine/tenofovir alafenamide (B/F/TAF) is a single-tablet regimen with high potency and good tolerability that can be used safely in multiple PWH populations, including treatment-naive PWH rapidly starting ART, and exhibits efficacy in PWH with a history of multiclass resistance. These features may facilitate immediate ART reinitiation and promote rapid VS among a broad population of PWH reengaging in care [14]. To successfully care for the PWH population with a history of poor retention, 2 critical steps in the care continuum deserve attention: (1) support mechanisms to reengage and retain in care and (2) immediate effective ART reinitiation. This may improve retention in care and accelerate VS, as observed in immediate ART models in treatment-naïve PWH [15–17]. The present study coupled rapid ART reinitiation with an intensive retention-in-care program to test the hypothesis that this approach may increase VS rates. Such a result may drive sustainability in preventing new HIV transmissions in a high-burden area to meet the EHE goals of reducing HIV as a public health threat.

## METHODS

### Study Design

This single-armed, single-site study (COMEBACK, NCT04519970) was conducted at the RMR CORE Center and was approved by the institutional review board of Cook County Health.

### Patient Consent

Participants provided written informed consent to all study procedures before enrollment.

### Study Population Selection

The RMR CORE Center has a low-barrier-care model, with many PWH presenting as walk-ins without preset appointments. PWH were recruited for this study if they presented to re-establish care in the clinic from September 2020 through October 2022. PWH were recruited through advertising in exam rooms, word of mouth, participant referral, and directly by health care providers. PWH were considered eligible if they were nonpregnant adults (≥18 years of age) off ART for ≥14 days at the time of enrollment, with no significant history of resistance-associated mutations to any of the components of B/F/TAF on prior resistance testing, no significant drug–drug interactions with B/F/TAF, and no history of recent renal impairment by an eGFR <30 mL/min (Table 1).

**Table 1.** Exclusion and Inclusion Criteria

Inclusion Criteria	Exclusion Criteria
HIV-seropositive	History of primary integrase inhibitor mutations, ≥3 TAMs, K70E, Q151M, T69 insertion, or combination of K65R + M184 V/I on prior resistance testing
Adult ≥18 y of age	Significant drug–drug interactions with B/F/TAF
Off ART ≥2 wk at time of enrollment	Pregnancy
History of eGFR ≥30 mL/min	Allergy to any component of B/F/TAF
Baseline labs (CBC, CMP, CD4+, HIV-1 RNA and resistance testing) collected at time of enrollment or <2 wk before enrollment	Signs or symptoms of opportunistic infection with cryptococcal meningitis, tuberculosis, or other infection that required delayed ART reinitiation
…	Unable/unwilling to provide consent

Abbreviations: ART, antiretroviral therapy; CBC, complete blood count; CMP, complete metabolic panel; eGFR, estimated glomerular filtration rate; TAMs, thymidine analog mutations.

### Outcomes

The primary objective was to determine VS rates and retention in care among PWH off ART who reengaged in care and were rapidly started on B/F/TAF with standard or enhanced CM. VS was defined as HIV-1 RNA <200 copies/mL using Ryan White (RW) Health Resources and Services Administration (HRSA) standards. The primary end point was the week 48 proportion of participants with VS in the intent-to-treat population. Retention in care was defined by the proportion of participants presenting to the week 4, 12, 24, and 48 study visits, respectively. Secondary end points were the proportions of participants with HIV-1 RNA cutoffs of <50 copies/mL at weeks 4, 12, 24, and 48; HIV-1 RNA <200 copies/mL at weeks 4, 12, and 24; and CD4+ cells/mm^3^ at weeks 24 and 48 in the intent-to-treat population and observed (missing [not retained at study time point] considered to be excluded) population.

Associations between non-VS at study end and the presence of any concerning social determinants of health (SDoH) at baseline were analyzed. SDoH assessed were housing insecurity, food insecurity, low health literacy, adherence concerns, active substance use, internalized stigma with disclosure concerns, and request for mental health services.

### Study Protocol

Eligibility information was collected from possible participants by study staff. PWH provided contact information and underwent a detailed structured interview to obtain demographic information, medical, HIV, and treatment history. If deemed eligible for the study, they completed 12 patient-related outcomes assessments with assistance of the retention specialist to identify factors that may impact their health management and treatment, that is, their SDoH. A comprehensive metabolic panel (CMP), CD4+/CD8+, HIV-1 RNA, and population-based RNA-1 genotype sequencing for resistance testing were completed at screening, B/F/TAF was then dispensed to participants with instructions for use, and follow-up appointments were made.

Clinical staff reviewed baseline lab values before the first follow-up visit. Participants were instructed to return to the clinic in 4 weeks for HIV-1 VL testing, and resistance data from the intake appointment were reviewed. They were then followed at weeks 12, 24, 36, and 48. At each appointment, participants underwent the following: medication reconciliation and review, targeted physical exam as indicated, adverse event assessment, blood draw (CMP, CD4+/CD8+, and HIV-1 RNA), participant self-report of adherence, and pill count for drug accountability. Refills of B/F/TAF were distributed at these study visits. In compensation, participants received $50 at baseline, week 24, and week 48 (study visits that included patient-related outcomes surveys) and $25 at weeks 4, 12, and 36. Participants were simultaneously encouraged to continue accessing their routine primary care at RMR CORE during their COMEBACK enrollment, and study staff worked closely with primary care providers to ensure access to care. In February 2023, due to feasibility and the absence of prespecified week 36 end points, the protocol was modified to exclude the week 36 study visit; the study was fully enrolled, all participants had surpassed the week 24 time point, and 85 participants had reached the week 36 time point. Study CM retention services and B/F/TAF refills were not affected.

### Case Management

**Figure 1.**
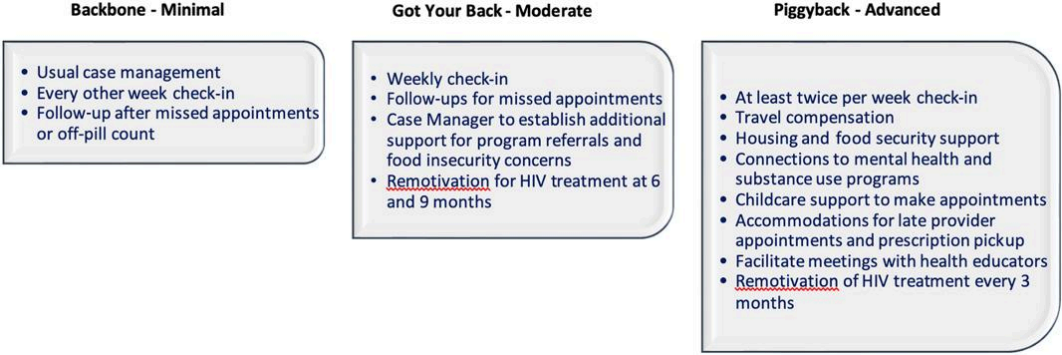
Case management tiers.

At enrollment, participants were screened into CM retention tiers: minimal, moderate, or advanced (Figure 1). Participants self-screened into their baseline tier based on their strengths and perceived ability to attain needed resources such as transportation, medical coverage, housing, mental health treatment, and substance use disorder treatment (Supplementary File 1).

We developed an acuity assessment tool based on number of participant contacts with the retention specialist, medication and study visit adherence, ability to complete study components, lab results (ie, sexually transmitted infections, coronavirus disease 2019 [COVID-19]), and other identified challenges, including concurrent health issues, to determine whether tier escalation was needed (Supplementary File 2). The tier level of CM support as the study progressed was reassessed for each participant at weeks 24 and 48 using the acuity assessment tool. The tier of CM support would be escalated if indicated, with increased communication, interim visits, additional resources, and/or additional support.

### Statistical Analysis

For a VS rate estimated at 75%, there is >90% power for a sample size of 100 PWH with a 2-sided Wilson score CI <0.05 for the primary outcome to exclude a VS rate of 50% or lower as a historical comparator approximated from recent HPTN 078 and CTN 049 studies [13, 18], accounting for contingencies for participant withdrawal and loss to follow-up [19]. All analyses for factors associated with VS at week 48 were performed using chi-square and Fisher exact tests as appropriate using Stata 18 (StataCorp LLC, College Station, TX, USA).

## RESULTS

### Baseline

One hundred PWH were enrolled into the COMEBACK study. Table 2 outlines the baseline demographic characteristics of both COMEBACK study participants and the general RMR CORE Center population prescribed B/F/TAF from 2020 to 2021. At baseline, study participants' median time off ART (range) was 5 (0.5–242) months, and the median VL (range) was 11 084 (40–2 000 000) copies/mL; 16% of participants exhibited VS at baseline with a VL <200 copies/mL. The median baseline CD4+ count (range) was 307 (6–1624) cells/mm^3^. The most recent ART prescribed before enrollment is shown in Table 2.

**Table 2.** Baseline Demographics

Demographics	General CORE Population on B/F/TAF 2020–2021 (n = 2212)	%	COMEBACK Study (n = 100)	%
Gender				
Cis-gender male, No.	1637	74	68	68
Cis-gender female, No.	555	25.1	24	24
Transgender male to female, No.	19	0.9	6	6
Transgender other/nonbinary, No.	1	0	2	2
Race and ethnicity				
Black/African American, No.	1345	60.8	90	90
Hispanic/Latinx, No.	644	29.1	5	5
White/Caucasian, No.	177	8	3	3
Other, No.	35	2.1	2	2
Age, y				
Median age (range), y	…	…	37 (24–68)	…
24–35, No.	497	22.5	49	49
36–47, No.	605	27.4	27	27
48–59, No.	722	32.6	16	16
60–68, No.	388	17.5	8	8
Baseline characteristics				
Median VL (range), copies/mL	…	…	11 084 (≤40–2 000 000)	…
VL <200 copies/mL, No.	…	…	16	…
Median time off ART (range), mo	…	…	5 (0.5–242)	…
Median No. of CD4+ T cells/mm^3^ (range)	…	…	307 (6–1624)	…
Last ART before enrollment				
B/F/TAF, No.	…	…	61	…
E/c/F/TAF, No.	…	…	14	…
ABC/3TC/DTG, No.	…	…	4	…
TAF/FTC + DTG, No.	…	…	3	…
Other, No.	…	…	18	…

Abbreviations: 3TC, lamivudine; ABC, abacavir; ART, antiretroviral therapy; B, bictegravir; C, cobicistat; DTG, dolutegravir; E, elvitegravir; F, emtricitabine; FTC, emtricitibine; TAF, tenofovir alafenamide; VL, viral load.

**Figure 2.**
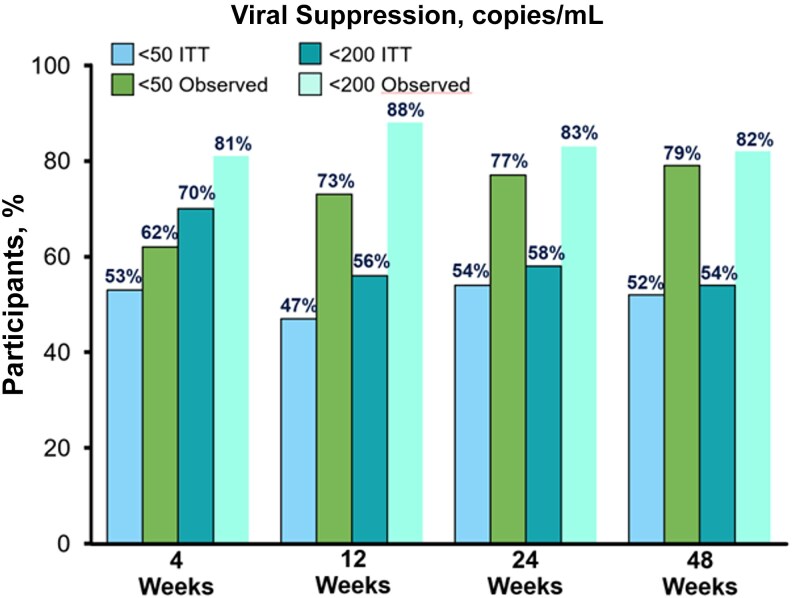
VS rates. Percentage of participants exhibiting VS at <50 and <200 copies/mL in the ITT (n = 100) and observed (week 4 n = 86, week 12 n = 64, week 24 n = 70, week 48 n = 66) study population analyses. Abbreviations: ITT, intent to treat; VS, viral suppression.

Figure 2 outlines the VS rates (at both <50 copies/mL and <200 copies/mL) in study participants by analyses of both the intent-to-treat and observed populations.

Sixty-six of 100 participants (66%) were retained in care at 48 weeks, with VS in 54% (n = 54/100) of the intent-to-treat population and 82% (n = 54/66) of the observed population. No resistance to B/F/TAF was detected through 48 weeks among participants who did not achieve VS and underwent population-based genotype resistance testing. There were no serious adverse events associated with B/F/TAF. The median CD4+ at week 24 was 432 cells/mm^3^, and it was 474 cells/mm^3^ at week 48, an increase of 125 and 167 cells/mm^3^ from baseline, respectively.

At week 4 of the study, 86% of participants were retained, 64% at week 12, 70% at week 24, and 66% by the last study visit, week 48. Loss to follow-up was the most common reason for study discontinuation (Table 3). Of those lost to follow-up, 9 did not respond to the retention specialist's repeated attempts at contact, while the phone numbers of an additional 9 were disconnected.

**Table 3.** Study Discontinuations Through Week 48 Including Lost to Follow-up

Discontinuation	No.
Screen failure: baseline eGFR = 19	1
Moved away	4
Death	3
Opioid overdose	2
Motor vehicle accident	1
Incarcerated	3
Switched to LA CAB/RPV	2
Pregnancy	1
Nonadherent, request to switch back to ABC/3TC/DTG	1
Nonadherent due to mental health and homelessness	1
Lost to follow-up	18
No response to retention specialist	9
Phone disconnected	9

Abbreviations: ABC/3TC/DTG, Abacavir/Lamivudine/Dolutegravir; eGFR, estimated Glomerular Filtration Rate; LA CAB/RPV, Long-Acting Cabotegravir/Rilpivirine.

Participants self-screened into minimal (70%), moderate (28%), and advanced (1%) CM tiers. One participant did not complete the baseline CM screening. Of the intent-to-treat population, 59% (59/100) required tier escalation during the study. The greatest proportion of transitions occurred from minimal at baseline to advanced (40%) by study end. Escalation of least 1 CM tier (*P* = .02) and presence of adherence concerns at baseline (*P* = .04) were significantly associated with non-VS at week 48 (Table 4).

**Table 4.** Factors Associated With VS at Week 48

Characteristic	Overall, No.	VS (<200 c/mL) = 54, No. (%)	Non-VS = 46, No. (%)	*P* Value
Gender				
Cis-gender male	68	36 (67)	32 (70)	.33
Cis-gender female	24	14 (26)	10 (22)	.59
Transgender M to F	6	2 (4)	4 (7)	.30
Transgender other/nonbinary	2	2 (4)	0 (0)	.19
Age ≥30 y	80	42 (78)	38 (83)	.33
Age ≥35 y	55	32 (59)	23 (50)	.40
Race/ethnicity				
Black/African American	90	49 (91)	41 (89)	.39
Hispanic/Latinx	5	3 (6)	2 (4)	.66
White/Caucasian	3	1 (2)	2 (4)	.25
Other	2	1 (2)	1 (2)	.91
Baseline VS (<200 copies/mL)	16	10 (19)	6 (13)	.55
Baseline CD4 ≥350 cells/mm^3^	40	24 (44)	16 (35)	.28
Baseline minimal CM	70	40 (74)	30 (65)	.33
Baseline mod-advanced CM	29	14 (26)	15 (33)	.48
Escalated at least 1 CM tier	59	21 (39)	38 (83)	.02
Housing insecurity	46	23 (43)	23 (50)	.88
Food insecurity	35	18 (33)	17 (37)	.84
Low health literacy	25	12 (22)	13 (28)	.24
Adherence concerns	33	14 (26)	19 (41)	.04
Active substance use	22	11 (20)	11 (24)	.98
Request for mental health services	23	11 (20)	12 (26)	.50
Internalized stigma with disclosure Concerns	23	11 (20)	12 (26)	.50
Baseline SDoH ≥1	85	45 (83)	40 (87)	.54
Baseline SDoH ≥2	63	30 (56)	33 (72)	.13
Baseline SDoH ≥3	36	18 (33)	18 (39)	.55
Baseline SDoH ≥4	19	9 (17)	10 (22)	.56

## DISCUSSION

The COMEBACK Study reengaged treatment-experienced PWH not on ART to immediately reinitiate ART with B/F/TAF to assess the impact of a retention specialist intervention stratified by self-reported needs on continuity of care and VS. With a baseline median time off ART of 5 months, at week 48, 54% (54/100) of reengaged participants (intent-to-treat population) achieved VS. While lower than the observed general PWH population at our center, this percentage represents incremental success in achieving virologic control for this lost-to-care population. Moreover, we observed 82% (54/66) VS among participants retained in care at week 48, which approaches national Health Resources and Services Administration figures for Ryan White clinics. Retention and virologic response rates were greatest at week 4, likely reflecting initial acceptance of our tailored CM model for rapid ART reinitiation coupled with accelerated viral decay kinetics associated with bictegravir [20]. There is public health benefit in reintroducing ART immediately upon reengagement in care to mitigate risk of HIV transmission [15–17]. B/F/TAF was well tolerated, with no drug-related adverse events greater than grade 2, no drug-related B/F/TAF discontinuations, and no resistance-associated mutations detected to B/F/TAF components during the study period.

The VS and retention rate end points in our study were less than contemporaneous Ryan White clinic data, primarily attributed to participants with missing data or discontinuation by study end (34%), despite enhanced CM. Nearly two-thirds of participants overall reported at least 2 SDoH at baseline, with adherence concerns at enrollment significantly associated with non-VS [21]. The existence of multiple SDoH can amplify challenges in ART adherence and result in missed medical appointments, which as an indicator of retention is an informative metric in identifying PWH at risk for poor health outcomes [22]. Missed visits have been associated with virologic nonsuppression [22, 23], decreased ART adherence [24], low CD4 count [25], AIDS-defining illnesses [26, 27], and mortality [25, 26]. It is possible that the tier level assessments contributed little value to reengagement in care; among the one-third of participants in our study who did not complete the week 48 visit end point, a majority (53%; 18/34) were lost to follow-up, with either no response to multiple phone calls and/or text reminders or the phone number no longer operable, which led to a subsequent breakdown in retention services. Several studies that have introduced increased personal contact with PWH supplemented through phone call and text messaging interventions have supported improved retention and adherence [28–30]. These systems, however, are predicated on continued access to mobile phones, which can be tenuous among PWH with low socioeconomic status [24, 31, 32], behavioral health problems [31], or poor social–cognitive resources [31]. Earlier studies have reported distinct disparities in cell phone access in urban PWH cohorts [33, 34]. Though cell phone ownership in the 2020s is universally high across a wide range of sociodemographic groups in the United States, at 96% among Black and 97% among White populations, smartphone usage drops to 84% among Black compared with 91% among White persons, and further declines to 79% in persons with incomes <$30 000 [35]. In our study, 90% of participants were Black, and >70% of our clinic population either is uninsured or receives government-assisted insurance, for which being at or below 138% of the Federal Poverty Level is a requirement. Regardless of race, financial constraints appear to be the greatest factor in maintaining smartphone services, with 48% of smartphone-dependent individuals in the United States facing cancellation of their mobile plans or simply shutting off their phones due to financial factors [36].

In low-barrier-care models, incentives encourage PWH to capitalize on care visits and help address competing needs [12, 37]; these incentive programs should prioritize smartphone distribution packaged with continuous service plans to enable unencumbered communication with the care team. In our study, there was agreed-upon privacy between the retention specialist and participant to assure comfort and personal information compliance, with collateral benefits beyond appointment reminders, including health-related messages such as result notification for VS that likely further promoted remotivation for optimal oral ART adherence behaviors. Even as long-acting ART is emerging as a viable option to achieve virologic control among PWH populations with a history of nonadherence to oral ART [38, 39], with 2 participants in our trial transitioning during the study to long-acting ART following adherence and VS with B/F/TAF, intensive ongoing social support and appointment reminder interventions remain critical, particularly relevant given the importance of on-time injection.

Our acuity assessment tool for CM and retention services determined that 59% of participants required tier escalation by study end, with the greatest proportion (40%) of transitions occurring from minimal at baseline to advanced. This may signal self-reported undervaluation of the support needed when coming back to care. In the United States, differentiated service delivery (DSD) aligns health resources for PWH with the greatest need to address complex barriers to care, including poverty, unstable housing, substance use, and behavioral health disorders [40, 41]. Modeling data have demonstrated that DSD can be cost-effective and increase life expectancy [42]. The tempo of usual CM at the RMR CORE Center consists of phone contact with PWH at least monthly, face-to-face contact at least every 3 months, and care planning and conferencing every 6 months. The COMEBACK study leveraged existing CM and social services at the RMR CORE Center with enhanced low-barrier care. This was exemplified by a personal retention specialist, who through frequent check-ins with patients at least weekly to biweekly coordinated both study visits and usual clinical care encounters via scheduled appointments and on a walk-in basis, delivered seamless access to ART, integrated care and referrals with RMR CORE case managers for multisector agencies interfacing with patients, and assisted patients in health literacy and psychosocial support using motivational interviewing, with a particular focus on medication adherence. The flexible tiered protocol design allowed for rapid modification of the intervention based on dynamic changes in each participant's SDoH needs and interim self-reported outcomes. Our intervention supports the feasibility of a dedicated retention specialist at a ratio of approximately 1 staff to 100 PWH at high risk for ART nonadherence and poor engagement in care. CM and retention resources escalation and low adherence self-efficacy at baseline were associated with non-VS in this analysis, suggesting that even among PWH who required or requested higher level CM tiers, detrimental SDoH can be entrenched and may impede successful adherence behaviors. Further resources targeted to each SDoH may be beneficial, at least for the first 12 months after reengagement in care.

### Study Limitations

The timing of our study may have led to limitations in ongoing engagement in care. Enrollment started in September 2020 when COVID-19 precautions at our clinic were still intact and continued until early summer of 2021. By participant reports to staff, additional barriers to clinic visits included reluctance in taking public transportation and restrictions in bringing children to appointments, increasing the already substantial burden of childcare. Participants attending appointments also had to adhere to the clinic's strict hand hygiene, social distancing, and mask-wearing protocols; the enforcement of these policies could lead to tension between clinic and research staff and participants. In addition, we selected 7 SDoH to assess as potential factors influencing VS; however, there may be other sociodemographic elements exerting barriers to retention in care and virologic control. In addition, due to the individualized CM provided by the retention specialist, our study findings may not be generalizable to people who do not speak English because our retention specialist was a monolingual English speaker.

### CONCLUSIONS

VS on B/F/TAF in the COMEBACK study was achieved in 54% by intention-to-treat, and 82% for participants retained in care at week 48, among treatment-experienced PWH off ART with SoDH challenges who immediately reinitiated ART with B/F/TAF, approaching national rates for all PWH with retention in Ryan White clinics. In addition, these participants experienced a progressive increase in CD4+ cells through 48 weeks. A majority of participants required escalation of CM resources, with lapses in retention among one-third of participants by study end, predominantly driven by disrupted smartphone communications. The findings demonstrate that achieving VS among high-risk PWH populations disenfranchised from care will likely require not only individualized case management and specialized retention approaches but integrated sustainable strategies among multiple levels of social determinants of health interventions to advance low-barrier-care models. Combined with immediate reinitiation of potent ART, these tools in EHE may help improve the cascade of care in the United States, prevent onward HIV transmission, and enhance overall quality of life for PWH.

## Supplementary Material

ofag216_Supplementary_Data

## Supplementary Data

Supplementary materials are available at *Open Forum Infectious Diseases* online. Consisting of data provided by the authors to benefit the reader, the posted materials are not copyedited and are the sole responsibility of the authors, so questions or comments should be addressed to the corresponding author.
