# Supplementary material for: The COMEBACK Study: Social Determinants of Health Impact on Virologic Suppression in a 48-Week Low-Barrier-Care Study of Rapid Antiretroviral Therapy Reinitiation Among Persons With HIV Lost to Care
Source: Open Forum Infect Dis. 2026 Apr 18;13(5):ofag216. doi: 10.1093/ofid/ofag216 (PMC13126515; doi:10.1093/ofid/ofag216)
Supplement: ofag216_Supplementary_Data [file ofag216_supplementary_data.zip › COMEBACK Manuscript Supplementary File 1.docx]

COMEBACK Study Screening Tool

DEMOGRAPHIC INFO

Name: ____________________________________________

Nickname:________________________________________

Gender Identity:

Cisgender Man☐ Cisgender Woman☐ Transgender Man☐ Transgender Woman☐ Non-binary☐ Other Identity:_______________

Pronouns:

He/him☐ She/her☐ They/them☐ Other Pronouns:_____________

Racial Identity (check all applicable):

Black or African American☐ Latino or Hispanic☐ White☐

Native American☐ Asian or Pacific Islander☐

Age _____________

Phone number ( ) ___ ___ ___ - ___ ___ ___

Email:____________________________________________________

Address:

Street ___________________________________________________

City__________________ Zip Code__________________________

Do you have regular and reliable access to a cell phone?

Yes☐ No☐

Do you have regular and reliable access to a computer?

Yes☐ No☐

Do you prefer to communicate through text, call, or email?

Text☐ Call☐ Email☐

Is it okay to leave a voicemail if I cannot reach you upon calling?

Yes, leave detailed voicemail☐ Yes, leave voicemail with no details☐

No☐

What is the highest level of education you have completed?

Middle School☐

High School☐

Associates Degree or Technical Training☐

Undergraduate Degree☐

Graduate Degree☐

STRENGTHS

1. What would you say some of your strengths are as…

A. An individual?

B. As a family member or friend?

C. As a worker?

D. As a community member?

2. Circle on the scales below how strongly you agree with the following statements.

A. I am an organized person.

Strongly Disagree Disagree Neutral Agree Strongly Agree

B. I am a creative person.

Strongly Disagree Disagree Neutral Agree Strongly Agree

C. I am a curious person.

Strongly Disagree Disagree Neutral Agree Strongly Agree

D. I love to learn.

Strongly Disagree Disagree Neutral Agree Strongly Agree

E. I make the right choices in difficult circumstances.

Strongly Disagree Disagree Neutral Agree Strongly Agree

F. I am a brave person.

Strongly Disagree Disagree Neutral Agree Strongly Agree

G. I am an honest person.

Strongly Disagree Disagree Neutral Agree Strongly Agree

H. I persevere through challenges.

Strongly Disagree Disagree Neutral Agree Strongly Agree

I. I am a kind person.

Strongly Disagree Disagree Neutral Agree Strongly Agree

J. I am a loving person.

Strongly Disagree Disagree Neutral Agree Strongly Agree

K. I build strong relationships with those around me.

Strongly Disagree Disagree Neutral Agree Strongly Agree

L. I am a leader.

Strongly Disagree Disagree Neutral Agree Strongly Agree

M. I value teamwork.

Strongly Disagree Disagree Neutral Agree Strongly Agree

N. I treat myself fairly.

Strongly Disagree Disagree Neutral Agree Strongly Agree

O. I treat others fairly.

Strongly Disagree Disagree Neutral Agree Strongly Agree

P. I can forgive myself for mistakes.

Strongly Disagree Disagree Neutral Agree Strongly Agree

Q. I am a humble person.

Strongly Disagree Disagree Neutral Agree Strongly Agree

R. I am funny.

Strongly Disagree Disagree Neutral Agree Strongly Agree

S. I am a hopeful person.

Strongly Disagree Disagree Neutral Agree Strongly Agree

T. I am either spiritual or religious.

Strongly Disagree Disagree Neutral Agree Strongly Agree

U. I express gratitude when I feel it.

Strongly Disagree Disagree Neutral Agree Strongly Agree

V. I ask for help when I need it.

Strongly Disagree Disagree Neutral Agree Strongly Agree

HEALTH AND ROUTINES

3. How long has it been since you were diagnosed with HIV?

Within the past 2 years☐ Between 2 and 5 years ago☐

Between 5 and 10 years ago☐ More than 10 years ago☐

4. When did you last receive treatment for HIV?

Within the past month☐ 2-3 months ago☐ 4-6 months ago☐

6 months to 1 year ago☐ 1-2 years ago☐ More than 2 years ago☐

5. Which most accurately describes your maintenance with medication routines?

I usually miss important medications.☐

I often miss important medications.☐

I take my medications sporadically.☐

I rarely forget to take my medications.☐

I always take my medications.☐

6. Which most accurately describes your attendance with doctor’s appointments?

I usually miss appointments.☐

I often miss appointments.☐

I make some of my appointments.☐

I make most of my appointments.☐

I make all of my appointments.☐

7. Does your daily schedule allow you to have a routine?

Yes☐ No☐

Please explain why yes or no: ___________________________________________________

8. What are some of the challenges you have had taking your medication or coming in for doctor’s visits in the past?

Insurance☐ Travel☐ Childcare☐ Work and Scheduling☐

Other:______________________________________________________________

9. How would you describe your personal style of communication?

A. Is it important to you that your doctor is friendly and talkative when communicating with you?

Not Important☐ Somewhat Important☐ Very Important☐

B. Is it important to you that your doctor is direct and to the point when communicating with you?

Not Important☐ Somewhat Important☐ Very Important☐

10. Why is treating your HIV important to you?

11. Do you have health insurance?

Yes☐ No☐

Since the COVID-19 (Coronavirus) pandemic became active in the US in February…

12. Have you been screened for COIVD-19?

Yes☐ No☐

Did you test positive?

Yes☐ No☐

13. Has COVID-19 affected your mental health? (Anxiety, stress, fear, etc.)

Yes☐ No☐

14. Has COVID-19 affected your ability to…

A. Get or take your medications Yes☐ No☐

B. Attend doctor’s appointments Yes☐ No☐

15. Have you been doing telehealth appointments? Yes☐ No☐

SUPPORTS

16. Do you have people in your life you have told about your HIV status? About how many people would you say you have disclosed your HIV status to?

Yes, 2 or more☐ Yes, 1☐ No☐

A. Are they individuals you feel comfortable asking for help?

Yes☐ No☐

B. Do you want to provide the contact info of any of these members of your support system? They would only be contacted in the case of an emergency or if we were unable to contact you for longer than a 2-week period.

Name:

Relation:

Phone:

Email:

Name:

Relation:

Phone:

Email:

17. Are you part of others’ support system?

Yes☐ No☐

18. Are you anyone’s primary caretakers?

Yes☐ No☐

19. What do you think will be the most helpful way I can add additional support for you in terms of your healthcare? (check all that apply)

Provide reminders for appointments☐

Someone to ask questions to☐

Emotional support and checking in☐

Other:

20. Are there any other resources for problems outside of your HIV care that you would like me to help connect you with?

Housing☐

Employment support☐

Mental healthcare☐

Other:

21. Do you have a case manager at the CORE Center?

Yes☐ No☐

If yes, who? ________________________________________________

COMEBACK Study Screening Tool Scorecard

| Question | Response | Point |
| --- | --- | --- |
| Age | Older than 50  Between 30 and 50  Younger than 30 | 0  1  2 |
| Education | Undergraduate or graduate degree  Associates degree or technical training  High school or middle school | 0  1  2 |
| 1 | Identifies more than 1 strength for A-D  Identifies at least 1 strength for A-D  Does not identify a strength for all 4 subparts | 0  1  2 |
| 2  Subparts each have their own score here (Between 0 and 16 points possible in the subparts)  Affirmative = Agree and strongly agree | A,E,G,H,M,P,S,V  Strongly Agree or Agree  A,E,G,H,M,P,S,V  Neutral  A,E,G,H,M,P,S,V  Disagree or Strongly Disagree  15 or more responses in the affirmative  Between 8 and 14 responses in the affirmative  7 or fewer responses in the affirmative | 0  1  2  0  1  2 |
| 3 | Within the past 2 years  More than 2 years ago | 0  1 |
| 5 | Always take or rarely forget medications  Sporadically take medications  Often or usually miss medications | 0  1  2 |
| 6 | Make all or almost all appointments  Make some appointments  Often or usually miss appointments | 0  1  2 |
| 7 | Yes  No | 0  1 |
| 8 | No challenges  1-2 challenges  More than 2 challenges | 0  1  2 |
| 10 | Articulates clear reason  Articulates some reason | 0  1 |
| 11 | Yes  No | 0  1 |
| 16 | Yes, 2 or more  Yes, 1  No   1. Yes   No | 0  1  2  0  1 |
| 20 | Asks for no additional resources  Asks for additional resources in one issue area  Asks for additional resources in multiple issue areas | 0  1  2 |
| 21 | Yes  No | 0  1 |

If individuals already have a case manager at CORE, they will not be placed in the Piggyback tier, unless explicitly requested or if they fall above 35 in the scoring. If individuals do not have health insurance, they are unable to be placed in the Backbone tier until they get health insurance or have a plan to with the Benefits Department, unless their score is 5 or below. Individuals are welcome to move into a tier of more intensive support if desired.

Lowest Possible Score: 0

Highest Possible Score: 40

0-13 = Backbone

14-26 = Got Your Back

27-40 = Piggyback
