# Supplementary material for: The COMEBACK Study: Social Determinants of Health Impact on Virologic Suppression in a 48-Week Low-Barrier-Care Study of Rapid Antiretroviral Therapy Reinitiation Among Persons With HIV Lost to Care
Source: Open Forum Infect Dis. 2026 Apr 18;13(5):ofag216. doi: 10.1093/ofid/ofag216 (PMC13126515; doi:10.1093/ofid/ofag216)
Supplement: ofag216_Supplementary_Data [file ofag216_supplementary_data.zip › COMEBACK Manuscript Supplementary File 2.docx]

**Adapted COMEBACK Acuity Tool**

**Patient Name** **________________________** **MRN ____________________**

**Date of Enrollment ____________________** **Participant Number ________**

**Enrollment Tier Placement __________________** **Study Completion Tier _____________**

|  | **1** | **2** | **3** | **4** |
| --- | --- | --- | --- | --- |
| 1. Medication Adherence | 100% - 76% | 75% - 51% | 50% - 26% | 25% or lower |
| 1. Study Visit Adherence | Attends all appointments (6). | Attends majority of appointments (4-5). | Attends some appointments (2-3). | Attends minimal appointments (1). |
| 1. Additional Outside Resources/Support | Needs no additional support or resources. | Needs little to no additional support or resources. | Needs some additional support or resources. | Needs significant additional support or resources. |
| 1. Study Components   **Note: This speaks to patient’s ability to complete steps of study at time of visit. | Was able to complete all components of study visit. | Was able to complete majority of components required for study visit | Was able to complete some/minimal study components at study visit. | Could not complete any components of study visit. |
| 1. INT Visit | Patient required no INT visits. |  |  | Patient required INT visit(s). |
| 1. Identified Challenges | No challenges. |  |  | Concurrent challenges (I.e. Comorbidity, substance use, domestic violence, mental health problems, homelessness, incarceration, STI(s), COVID). |
| 1. Labs* | Completed the study undetectable. | Patients VL >1000.  CORE metrics establish all high risk ≥ 1000 | | Viral load uncontrolled. |
| 1. Communication and Contact | Patient does not require nor requested additional communication. | Patient requires or requests some additional communication. | | Patient requires or requested significant additional communication. |
| Total Score |  |  |  |  |

Notes: ___________________________________________________________________________________________________________________________________________________________________________________________________________________________________________________________________________________________________________________________________________________________________________________________________________________________________

Date of Review: ________________________________

Signature: _____________________________________

Acuity level guidelines for tier transition:

Once a tier escalation was determined, there was no reversion to a lower level of support.

Stable level 1: Continue Backbone (minimal) support

Any level 2: Got Your Back (moderate) support

Any level 3 or 4: Piggyback (advanced) support

*VL results were not used for tier reassessment during weeks 24 and 48 as these lab levels were drawn during the day of the respective study visits. The VL level was recorded in the acuity tool once available for future counseling if detectable levels correlated with medication adherence scores.
